# Supplementary material for: Physical activity and health-related quality of life among adults living in Jeddah city Saudi Arabia
Source: PeerJ. 2023 Sep 11;11:e16059. doi: 10.7717/peerj.16059 (PMC10501367; doi:10.7717/peerj.16059)
Supplement: Supplemental Information 2 [file peerj-11-16059-s002.docx]

**Key**

| **Sex** |  |
| --- | --- |
| Female | 1 |
| Male | 2 |
| **Nationality** |  |
| Non Saudi | 1 |
| Saudi | 2 |
| **Marital status** |  |
| Divorced | 1 |
| Married | 2 |
| Single | 3 |
| Widow | 4 |
| **Education** |  |
| Up to High school | 1 |
| Bachelor's/Diploma | 2 |
| Masters and above | 3 |
| **Age group** |  |
| 18-29 | 1 |
| 30-44 | 2 |
| 45-60 | 3 |
| > 60 | 4 |
| **Location** |  |
| Gym/ Walking tracks | 1 |
| Hospital visitors | 2 |
| Malls | 3 |
| **Smoking (cigarette or sheesha)** |  |
| No | 0 |
| Yes | 1 |
| **Comorbid condition** |  |
| No | 0 |
| Yes | 1 |
