# Supplementary material for: Physical activity and health-related quality of life among adults living in Jeddah city Saudi Arabia
Source: PeerJ. 2023 Sep 11;11:e16059. doi: 10.7717/peerj.16059 (PMC10501367; doi:10.7717/peerj.16059)
Supplement: Supplemental Information 3 [file peerj-11-16059-s003.docx]

**Supplementary table S1**

**Table S1: Calculating the 4-level physical activity index**

| **Physical activity level** | **Explantation** |
| --- | --- |
| Active | Sedentary job and ≥ 3 hours physical exercise and / or cycling  per week OR  Standing job and 1-2.9 hours physical exercise and / or cycling  per week OR  Physical job and some but < 1 hour physical exercise and / or  cycling per week OR  Heavy manual job |
| Moderately active | Sedentary job and 1-2.9 hours physical exercise and / or cycling  per week OR  Standing job and some but < 1 hour physical exercise and / or  cycling per week OR  Physical job and no physical exercise or cycling |
| Moderately inactive | Sedentary job and some but < 1 hour physical exercise and / or  cycling per week OR  Standing job and no physical exercise or cycling |
| Inactive | Sedentary job and no physical exercise or cycling |

**Table S2: Multivariate regression analysis (unadjusted and adjusted regression coefficient, β), evaluating factors associated with PCS and health-related quality of life among adults in Jeddah, n=693**

|  | **Physical component summary (PCS)** | | | |
| --- | --- | --- | --- | --- |
| **Characteristics, n %** | **Unadjusted analysis**  **(95% CI)** | **P-value** | **Adjusted analysis) (95% CI)** | **P-value** |
| Sex |  |  |  |  |
| Female | Reference |  |  |  |
| Male | 4.61 (1.73, 7.48) | 0.002 | 4.43 (1.44, 7.41) | 0.004 |
| Age group |  |  |  |  |
| 18-29 | Reference |  |  |  |
| 30-44 | -2.33 (-5.57, 0.90) | 0.157 | -4.06 (-7.87, -0.25) | 0.036 |
| 45-60 | -7.53 (-11.90, -3.16) | 0.001 | -6.27 (-11.50, -1.03) | 0.019 |
| > 60 | -16.5 (-24.83, -8.24) | <0.001 | -8.44 (-17.59, 0.71) | 0.071 |
| Nationality |  |  |  |  |
| Saudi | Reference |  |  |  |
| Non-Saudi | 1.44 (-2.92, 5.81) | 0.517 | - | - |
| Educational status |  |  |  |  |
| Up to High school | Reference |  |  |  |
| Bachelor's/Diploma | 1.89 (-1.51, 5.29) | 0.276 | 0.53 (-2.85, 3.91) | 0.758 |
| Masters and above | 4.54 (-0.23, 9.31) | 0.062 | 3.07 (-1.58, 7.72) | 0.196 |
| Marital status |  |  |  |  |
| Single | Reference |  |  |  |
| Married | -1.41 (-4.46, 1.63) | 0.058 | 1.51 (-2.15, 5.18) | 0.417 |
| Divorced | -6.02 (-12.25, 0.21) | 0.362 | -0.47 (-6.83, 5.98) | 0.896 |
| Widow | -19.63 (-31.66, -7.61) | 0.001 | -8.48 (-20.73, 3.77) | 0.175 |
| Location |  |  |  |  |
| Gym/ Walking tracks | Reference |  |  |  |
| Malls | -2.89 (-6.18, 0.38) | 0.084 | -0.80 (-3.99, 2.38) | 0.621 |
| Hospital visitors | -3.88 (-7.88, 0.10) | 0.056 | 0.21 (-3.69, 4.12) | 0.914 |
| Smoking (cigarette or sheesha) |  |  |  |  |
| No | Reference |  |  |  |
| Yes | -10.13 (-15.04, -5.23) | <0.001 | -5.52 (-10.47, -0.56) | 0.029 |
| Comorbid condition |  |  |  |  |
| No | Reference |  |  |  |
| Yes | -15.92 (-21.84, -9.99) | <0.001 | -11.21 (-17.39, -5.03) | <0.001 |
| Physical activity |  |  |  |  |
| Inactive | Reference |  |  |  |
| Moderately inactive | 2.88 (-1.66, 7.44) | 0.214 | 1.27 (-3.27, 5.82) | 0.583 |
| Moderately active | 9.20 (4.97, 13.43) | <0.001 | 5.73 (1.28, 10.17) | 0.012 |
| Active | 14.54 (10.16, 18.92) | <0.001 | 10.11 (5.44, 14.77) | <0.001 |

**Table S3: Multivariate regression analysis (unadjusted and adjusted regression coefficient, β), evaluating factors associated with MCS and health-related quality of life among adults in Jeddah, n=693**

|  | **Mental component summary (MCS)** | | | |
| --- | --- | --- | --- | --- |
| **Characteristics, n %** | **Unadjusted analysis (95% CI)** | **P-value** | **Adjusted analysis (95% CI)** | **P-value** |
| Sex |  |  |  |  |
| Female | Reference |  |  |  |
| Male | 7.86 (4.88, 10.83) | <0.001 | 6.51 (3.40, 9.63) | <0.001 |
| Age group |  |  |  |  |
| 18-29 | Reference |  |  |  |
| 30-44 | -1.19 (-4.63, 2.25) | 0.497 | -4.76 (-8.74, -0.78) | 0.019 |
| 45-60 | -1.12 (-5.76, 3.51) | 0.634 | -2.36 (-7.82, 3.09) | 0.396 |
| > 60 | -6.63 (-15.45, 2.18) | 0.140 | -2.68 (-12.24, 6.86) | 0.581 |
| Nationality |  |  |  |  |
| Saudi | Reference |  |  |  |
| Non-Saudi | -1.20 (-5.77, 3.37) | 0.606 | - |  |
| Educational status |  |  |  |  |
| Up to High school | Reference |  |  |  |
| Bachelor's/Diploma | -2.05 (-5.62, 1.50) | 0.257 | -1.62 (-5.16, 1.90) | 0.366 |
| Masters and above | 2.27 (-2.71, 7.27) | 0.371 | 1.56 (-3.29, 6.42) | 0.527 |
| Marital status |  |  |  |  |
| Single | Reference |  |  |  |
| Married | 2.34 (-0.86, 5.54) | 0.151 | 3.92 (0.09, 7.74) | 0.045 |
| Divorced | -5.37 (-11.93, 1.17) | 0.108 | -0.16 (-6.85, 6.52) | 0.962 |
| Widow | -6.00 (-18.64, 6.63) | 0.351 | 1.22 (-11.55, 14.01) | 0.851 |
| Location |  |  |  |  |
| Gym/ Walking tracks | Reference |  |  |  |
| Malls | -1.14 (-4.58, 2.30) | 0.515 | 0.91 (-2.41, 4.23) | 0.591 |
| Hospital visitors | -3.54 (-7.73, 0.63) | 0.097 | 1.39 (-2.68, 5.47) | 0.502 |
| Smoking (cigarette or sheesha) |  |  |  |  |
| No | Reference |  |  |  |
| Yes | -7.55 (-12.72, -2.39) | 0.004 | -4.03 (-9.20, 1.13) | 0.126 |
| Comorbid condition |  |  |  |  |
| No | Reference |  |  |  |
| Yes | -13.72 (-19.96, -7.47) | <0.001 | -13.08 (-19.52, -6.63) | <0.001 |
| Physical activity |  |  |  |  |
| Inactive | Reference |  |  |  |
| Moderately inactive | -1.28 (-6.03, 3.45) | 0.594 | -1.60 (-6.35, 3.13) | 0.506 |
| Moderately active | 9.21 (4.80, 13.63) | <0.001 | 7.57 (2.92, 12.21) | 0.001 |
| Active | 12.81 (8.24, 17.38) | <0.001 | 9.77 (4.90, 14.64) | <0.001 |
