# Supplementary material for: Physical activity and health-related quality of life among adults living in Jeddah city Saudi Arabia
Source: PeerJ. 2023 Sep 11;11:e16059. doi: 10.7717/peerj.16059 (PMC10501367; doi:10.7717/peerj.16059)
Supplement: Supplemental Information 4 [file peerj-11-16059-s004.pdf]

How to Score the Rand SF-36 Questionnaire

STEP 1: SCORING QUESTIONS:

QUESTION NUMBER	ORIGINAL RESPONSE	RECORDED VALUE
1, 2, 20, 22, 34, 36	1	100
	2	75
	3	50
	4	25
	5	0
3, 4, 5, 6, 7, 8, 9, 10, 11, 12	1	0
	2	50
	3	100
13, 14, 15, 16, 17, 18, 19	1	0
	2	100
21, 23, 26, 27, 30	1	100
	2	80
	3	60
	4	40
	5	20
	6	0
24, 25, 28, 29, 31	1	0
	2	20
	3	40
	4	60
	5	80
	6	100
32, 33, 35	1	0
	2	25
	3	50
	4	75
	5	100

STEP 2: AVERAGING ITEMS TO FORM 8 SCALES:

SCALE	NUMBER OF ITEMS	AFTER RECORDING AS PER TABLE 1, AVERAGE THE FOLLOWING ITEMS
**Physical functioning**	10	3, 4, 5, 6, 7, 8, 9, 10, 11, 12
**Role limitations due to physical health**	4	13, 14, 15, 16
**Role limitations due to emotional problems**	3	17, 18, 19
**Energy/ fatigue**	4	23, 27, 29, 31
**Emotional well being**	5	24, 25, 26, 28, 30
**Social functioning**	2	20, 32
**Pain**	2	21, 22
**General health**	5	1, 33, 34, 35, 36

STEP 3: FIGURING SCORES:

RAND recommends the following straightforward approach to scoring the RAND 36-Item Health Survey.

All questions are scored on a scale from 0 to 100, with 100 representing the highest level of functioning possible. Aggregate scores are compiled as a percentage of the total points possible, using the RAND scoring table (STEP I chart).

The scores from those questions that address each specific area of functional health status (STEP II chart) are then averaged together, for a final score within each of the 8 dimensions measured. (eg pain, physical functioning etc.)

For example, to measure the patients energy/fatigue level, add the scores from questions 23, 27, 29, and 31. If a patient circled 4 on 23, 3 on 27, 3 on 29 and left 31 blank, use table 1 to score them.

An answer of 4 to Q23 is scored as 40, 3 to Q27 is scored as 60, and 3 to Q29 is scored as 40. Q31 is omitted. The score for this block is 40+60+40 =140. Now we divide by the 3 answered questions to get a total of 46.7. Since a score of 100 represents high energy with no fatigue, the lower score of 46.7% suggests the patient is experiencing a loss of energy and is experiencing some fatigue.

All 8 categories are scored in the same way. Using this questionnaire at the beginning and during the course of care, we can track the progress of the 8 parameters mentioned in the STEP II chart. Pretty nifty, eh?
